# Superior mesenteric artery embolism after radiofrequency ablation in regularly anticoagulated patients with paroxysmal atrial fibrillation: a case report

**DOI:** 10.1186/s12872-023-03066-5

**Published:** 2023-01-30

**Authors:** Yongle Jing, Jianqiang Xu, Bingwei Chen, Dasheng Xia, Dachuan Xia, Yunpeng Tian, Wei Xia, Chengzhi Lu, Yuli Wu

**Affiliations:** 1 Department of Cardiology, Tianjin First Central Hospital, No. 24, Fukang Road, 300192 Tianjin, China; 2 Department of Anesthesiology, Tianjin First Central Hospital, No. 24, Fukang Road, Tianjin, 300192 China

**Keywords:** Atrial fibrillation, Radiofrequency ablation, Superior mesenteric artery embolism, Abdomen pain

## Abstract

**Background:**

Superior mesenteric artery embolism (SMAE) is a rare cause of acute abdomen, and the fatality rate is extremely high if it is not diagnosed and treated in time. Due to the lack of knowledge and experience of nonspecialist physicians, it is easy to misdiagnose. Radiofrequency ablation (RFA) has become the first-line treatment strategy for atrial fibrillation (AF). Thromboembolic events are some of the major complications after RFA, whereas SMAE is rarely reported.

**Case presentation:**

A 70 year-old woman with paroxysmal AF who regularly took anticoagulant drugs for 3 months experienced abdominal pain after RFA. At the outset, she was misdiagnosed as mechanical intestinal obstruction. When the patient presented with blood in the stool, abdominal enhancement computed tomography was conducted and showed a small bowel perforation. Immediate laparotomy was performed, and the final diagnosis was SMAE.

**Conclusion:**

It is suggested that for unexplained abdominal pain after RFA of AF, the possibility of SMAE should be considered, and a targeted examination should be carried out in time to confirm the diagnosis and give appropriate treatment.

## Background

Superiormesenteric artery embolism (SMAE) is one cause of acute abdomen. SMAE is caused by obstruction of the blood supply to the intestinal wall due to occlusion of the mesenteric blood vessels. Due to the lack of specific clinical manifestations, diagnosis and treatment are often delayed. The disease has an acute onset, rapid progression, and dangerous condition, which can easily lead to ischaemic intestinal necrosis and even death. Studies have shown that due to untimely diagnoses, the fatality rate can be as high as 70–90%, and early diagnosis and treatment can significantly improve the success rate of treatment [1].

Radiofrequency ablation (RFA) has become the first-line treatment strategy for atrial fibrillation (AF). However, intraoperative RFA procedures may cause the formation of intracardiac thrombi and increase the risk of perioperative embolism in patients with atrial fibrillation [2, 3]. The incidence of thromboembolism caused by AF ablation is 0.28–2.8% [2]. The prevention of thromboembolic events during the periprocedural period of AF ablation is extremely crucial. The current guidelines recommend at least 3 weeks of effective anticoagulation prior to ablation for patients with AF at intermediate or high thrombotic risk [4]. This article reports a case of a patient with paroxysmal AF who had regularly taken anticoagulants for 3 months but developed SMAE after RFA. We retrospectively analysed relevant clinical data and summarize the experiences and lessons, aiming to provide clinical help.

## Case presentation

A 70 year-old woman came to our hospital with intermittent wheezing for more than 10 days. She had a history of coronary heart disease for more than 3 years, and one stent was implanted in the first diagonal to the left anterior descending (LAD) after coronary angiography (CAG), which indicated 90% stenosis of the first diagonal opening. Three months prior, the patient had an acute anterior wall myocardial infarction. CAG indicated occlusion of the LAD, and another stent was implanted in the LAD. For more than 3 years, she had paroxysmal AF, and she was given clopidogrel 75 mg qd combined with rivaroxaban 15 mg qd for the treatment of paroxysmal AF in the prior 3 months. She had a history of chronic cardiac insufficiency, hypertension, type 2 diabetes, and renal insufficiency. On admission, acute heart failure was considered, and the symptoms were relieved after vasodilator and diuresis. The serum creatinine level of the patient on admission was 120 µmol/L (normal value: 45–84 µmol/L). Echocardiography showed that the left heart was enlarged (anteroposterior diameter of left atrium: 39 mm, end-diastolic diameter of left ventricular: 53 mm). The apical segment of the ventricular septum and the anterior wall of the left ventricle lose the ability to systolic or diastolic movement. The apex of the left ventricle moved paradoxically. The left ventricular ejection fraction was 30%. The patient had recurrent episodes of AF with a rapid ventricular rate after admission. The CHA2DS2-VASc score was 6, and the HAS-BLED score was 2. We administered clopidogrel combined with rivaroxaban for antithrombosis and amiodarone for antiarrhythmia. Left atrial or atrial appendage thrombus was excluded by left atrial CT, and RFA was then performed to treat her paroxysmal AF. The patient was in sinus rhythm before the operation. After the atrial septal puncture was completed under fluoroscopy, 7500 IU of unfractionated heparin was given according to 100 IU/kg. 3D electroanatomical mapping using the CARTO3 system (Biosense Webster) was performed. The power ablation mode was used, and the power was 40 W. Bilateral pulmonary vein isolation was performed by using a cold saline irrigated RFA catheter. During the ablation process, the pressure was controlled between 5 and 20 g. The activated clotting time (ACT) was 229 s at half an hour after the atrial puncture, followed by an additional 1000 IU of heparin. The RFA process was completed before the next ACT test. After the operation, 15 mg of rivaroxaban was administered orally.

After the patient had returned to the ward, she developed diffuse abdominal pain with abdominal distension, which had both persistent and paroxysmal exacerbations, and the patient developed frequent vomiting and dysuria. The abdominal examination showed that the abdomen was soft, and the upper abdomen was slightly tender. The patient’s bloating was slightly relieved after the placement of a urinary catheter. However, after being given acid suppression, antispasmodic and antiemetic treatment, the symptoms of abdominal pain and vomiting were not obviously relieved. At 12 h after RFA, the abdominal pain of the patient was slightly relieved, but there was mild tenderness in the upper abdomen, right abdomen, and around the umbilicus, no obvious muscle tension and no rebound tenderness, and active bowel sounds. Abdominal CT showed ileocecal thickening with upper small bowel obstruction, and enhanced CT was recommended. After that, we considered the possibility of mechanical small bowel obstruction due to an ileocecal mass. Because the patient had renal insufficiency and the left atrial CT was performed before RFA, we did not urgently check the abdomen with contrast-enhanced CT, which was not performed to reduce the occurrence of contrast agent nephropathy. Laboratory tests showed elevated white blood cell (WBC, 20.83 × 10^9^/L, normal value: 3.5–9.5 × 10^9^/L), neutrophil percentage (88.6%, normal value: 40–75%), and creatinine (130.00 µmol/L, normal value: 45–84 µmol/L), and normal levels of tumour markers. Abdominal ultrasonography revealed only an enlarged gallbladder with deposits and a small amount of fluid around the spleen. We instructed the patient to fast with water. The patient passed stool after the enema. Cefoperazone sodium and sulbactam sodium were prescribed for anti-infection, and the condition of the patient was continuously observed. At 36 h after RFA, laboratory tests showed elevated WBC (15.26 × 10^9^/L), neutrophil percentage (87.5%), C-reactive protein (CRP, 442 mg/L, normal value: 0–6 mg/L), and procalcitonin (4.62 ng/ml, normal value: 0–0.5 ng/ml). At 72 h after RFA, the abdominal pain was slightly relieved, but the bowel sound was weakened. In addition, blood in the stool appeared. Laboratory tests showed faecal occult blood3+ and elevated WBC (14.64 × 10^9^/L), neutrophil percentage (85.3%), CRP (408 mg/L), procalcitonin (6.37 ng/ml), D-dimer (2881.96 µg/L, normal value: 0–500 µg/L), and lactic acid (2.7 mmol/L, normal value: 1.0–1.7 mmol/L). Then, we performed a contrast-enhanced CT of the abdomen, which showed pelvic small bowel necrosis and thickening of the ileocecal and terminal ileal walls.

**Fig. 1:**
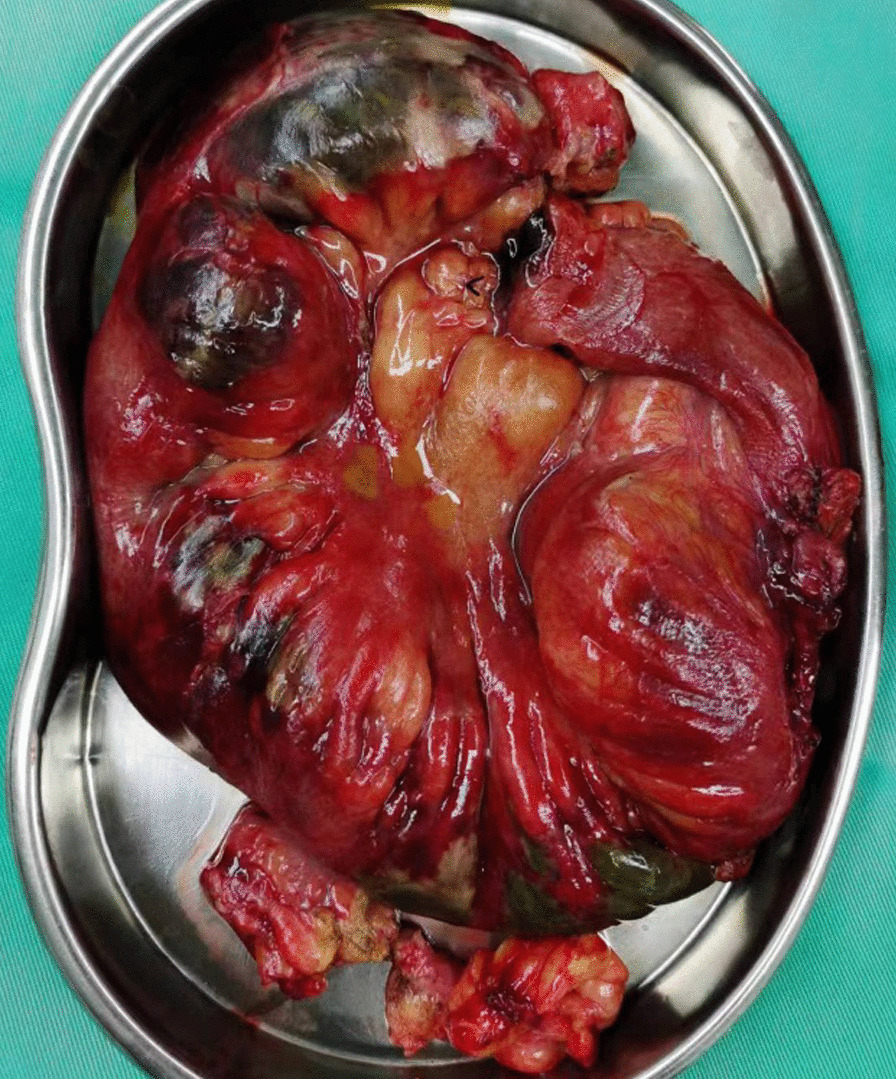
The ileum resected during operation

Laparotomy was performed immediately after the surgeon’s consultation. The exploration revealed dark red exudate in the pelvic cavity, and the amount was approximately 500 ml. Purulent material was attached to the surface of the small intestine. Thirteen to forty-five cm away from the ileocecal ileum, the colour of the ileum turned black, and the peristalsis disappeared, which was considered to be due to small intestinal necrosis. The contralateral side of the necrotic midsection mesentery was perforated. The perforation diameter was approximately 3 mm. The surgeon performed partial resection and anastomosis of the ileum, and 38 cm of the ileum was completely resected (Fig. 1). During the operation, the appendix was congested and oedematous, and the surface was covered with purulent material. An appendectomy was performed. Copious amount of warm saline was used to repeatedly rinse the abdominal cavity. After surgery, we administered cefoperazone sodium and sulbactam sodium combined with linezolid for anti-infection and parenteral nutrition support. Considering the possibility of SMAE after RFA, low molecular weight heparin was added for anticoagulation the next day after surgery. The samples of ascites collected during operation was cultured for common bacterial and showed the presence of Pseudomonas adaceae, Enterococcus faecalis, and Staphylococcus haemolyticus. Postoperative pathological examination confirmed a small bowel wall haemorrhagic infarction with inflammatory fibrinous exudation, vasodilation and congestion, and thrombosis. The postoperative diagnoses that were made were SMAE and partial ileal necrosis with perforation. On the 9th day after surgery, enhanced abdominal CT showed postoperative changes in the pelvic small intestine, and the thickening of the ileocecal and terminal ileal wall was better than before. After 1 month of treatment, the patient’s condition improved, and she was discharged. After one month of follow-up, the patient’s condition was stable.

## Discussion and conclusion

Thromboembolic events are some of the major complications after RFA, whereas SMAE is rarely reported. The opening of the main trunk of superior mesenteric artery (SMA) is large and has an oblique angle with the abdominal aorta, so the embolus from heart is easy to enter SMA. The formation of intracardiac thrombi during RFA is a result of multiple factors. Local overheating caused by electrical discharge during RFA results in local myocardial tissue damage and inflammation, which activate a coagulation cascade and promote platelet aggregation [3, 5]. Besides, although AF turns into sinus rhythm after RFA, left atrium function is impaired acutely following AF catheter ablation, resulting in slow blood flow in the left atrium, and thus prone to thrombosis in the left atrium [6, 7]. By using intracardiac echocardiography, Maleki et al. found that most of the intraoperative thrombus originated around the sheath placed in the left atrium during the operation and could form almost immediately after the sheath enters the left atrium [8]. Ren et al. reported that a 10% incidence of left atrium thrombus formation was detected on the sheath or mapping catheter during AF ablation [9]. Atrial thrombosis is significantly correlated with ACT. Gaita et al. showed that a low ACT (< 250 s) was an independent predictor of periprocedural thromboembolic events [10]. Therefore, achieving an effective anticoagulation level immediately after the sheath enters the left atrium is crucial to reducing perioperative thrombosis and embolic events. The guidelines recommend that the first loading dose of heparin should be injected immediately after the atrial puncture, and the intraoperative anticoagulation program should be combined with the patient’s body weight and ACT monitoring results to maintain an effective anticoagulation state at 300–350 s of ACT [11]. In this case, we stopped rivaroxaban preoperatively and administered unfractionated heparin at 100 IU/kg after the atrial septal puncture. Half an hour later, the ACT was 229 s, and 1000 IU of heparin was added. After RFA was completed, rivaroxaban was given instantly. We analysed the cause of the thromboembolic events in this case. It was most likely that the degree of anticoagulation during RFA did not meet the standard, resulting in atrial thrombosis. Therefore, we should closely monitor the ACT during RFA and add heparin according to the ACT results until the ACT reaches more than 300 s. If the ACT is not up to standard continuously, it is necessary to shorten the monitoring interval and increase the heparin dose. However, even if ACT achieves effective anticoagulation, embolic events may still occur. In addition to the embolism caused by the blood clot, there may be other emboli, including air, tissue, char formation, calcium, or foreign material formed during the procedure of RFA [3, 11]. Thus, despite the adoption of routine uninterrupted periprocedural anticoagulation, the days of concern about embolism during AF ablation are far from over.

Due to the short tolerance time of intestinal ischaemia and the lack of effective collateral circulation, SMAE can cause a patient’s condition to deteriorate rapidly. Diagnosis in the early stage of the disease, especially before intestinal necrosis, is the key to reducing the mortality rate. Older patients with AF, valvular heart disease, congestive heart failure, recent myocardial infarction, and bacterial endocarditis are at increased risk of SMAE. SMAE is characterized by a sudden onset of marked abdominal pain that is clearly disproportionate to the findings on physical examination. Abdominal pain is characterized by persistent, diffuse, or umbilical colic-like pain and is usually difficult to relieve with antispasmodic drugs. It is often accompanied by gastrointestinal emptying symptoms such as nausea, vomiting, and diarrhoea, without obvious peritoneal irritation. The pathophysiological process correlates well with the clinical scenario. In the first phase, the patient feels abdominal pain because of bowel ischaemia and hyperperistalsis. After 3–6 h, the second phase is followed by a painless interval due to damage to intramural pain receptors as a result of prolonged hypoperfusion. In the third phase, mucosal necrosis occurs, followed by infarction of the bowel wall. Bacterial translocation leads to gangrene of the intestine, diffuse peritonitis, sepsis, and multiorgan failure [12]. Selective digital subtraction angiography (DSA) is considered the gold standard for diagnosing SMAE. CTA is not only rapid, noninvasive, easily available, and highly accurate (sensitivity 89%, specificity 99%) in diagnosing mesenteric ischaemia but also helpful in detecting the underlying aetiology of ischaemia [13]. X-ray, colour Doppler ultrasound, and abdominal CT have low specificity and sensitivity in the diagnosis of mesenteric ischaemic disease and can be used as primary screening methods. The laboratory tests are not specific and can be manifested as increased white blood cells, D-dimer, amylase, and serum lactate levels and metabolic acidosis, which can reflect the severity of the patient’s condition to a certain extent. Intestinal necrosis is often suggested if paracentesis draws nonclotting fluid. If diagnosis is difficult, early exploratory laparotomy or laparoscopy is not only a reliable diagnostic method but also a treatment method [14].

Timely treatment is the key to improving the cure rate of SMAE patients. Blind observation or conservative treatment will cause the best time for treatment to be missed. Treatments for SMAE include general treatment, surgery or endovascular therapy [14]. In terms of drug therapy, anticoagulants, fluid replenishment increasing visceral perfusion, drugs relieving mesenteric vasospasm and broad-spectrum antibiotics should be immediately administered if drug contraindications are excluded [14]. Because of the extremely high mortality rate, drug therapy alone is not recommended, and early surgery or endovascular therapy is recommended [15]. Surgery is the traditional treatment modality for patients with SMAE, including simple incision and thrombectomy, simple intestinal resection, and thrombectomy combined with intestinal resection [15, 16]. Thrombus removal can be used to restore blood supply and avoid bowel resection or reduce the area of resection. Angioplasty and vascular bypass surgery can be considered for patients with unsatisfactory blood supply after thrombectomy [15]. If the bowel is ischaemic and necrotic, necrotic bowel resection should be performed as soon as possible. Endovascular therapy has gradually become another major treatment method for SMAE in recent years, and it includes catheter-directed thrombolysis, percutaneous thrombus aspiration, percutaneous mechanical thrombectomy, and percutaneous transluminal angioplasty [17, 18]. However, endovascular treatment has taken on much greater significance in patients without clinical signs of peritonitis, provided that the patient is haemodynamically stable [18]. Because of the inability to observe bowel status and resolve bowel necrosis, some patients undergoing endovascular therapy may need to be converted to surgical treatment. However, if a hybrid operating room is available, endovascular intervention can be selected in combination with laparotomy [18].

In conclusion, we need to give anticoagulant drugs in strict accordance with the treatment specifications in the perioperative period of RFA to reduce thromboembolic complications. If acute abdomen occurs after RFA, it is necessary to be highly vigilant about SMAE and conduct targeted examinations instantly. An unspecific routine diagnostic program or the wrong diagnostic tools extend the time to diagnosis and contribute to high mortality. Once the disease is diagnosed, it should be actively dealt with. The general principle is to relieve arterial obstruction as soon as possible, restore intestinal blood supply, and decide whether to perform intestinal resection according to the presence or absence of intestinal ischaemic necrosis.

## Data Availability

The data used in the case report are available from the corresponding author on reasonable request.

## Abbreviations

ACT: Activated clotting time
AF: Atrial fibrillation
CAG: Coronary angiography
CRP: C-reactive protein
DSA: Digital subtraction angiography
LAD: Left anterior descending
RFA: Radiofrequency ablation
SMAE: Superior mesenteric artery embolism
SMA: The superior mesenteric artery
WBC: White blood cell
